# Plasma Aryl Hydrocarbon Receptor Agonist Activity Is Associated With Inflammation and Metabolic Dysregulation in Obesity: A Cross‐Sectional Study

**DOI:** 10.1002/dmrr.70181

**Published:** 2026-06-07

**Authors:** Fatemah Bahman, Shihab Kochumon, Ashraf Al Madhoun, Areej Al‐Roub, Abdullah Bennakhi, Monira Arouj, Fahd Al‐Mulla, Rasheed Ahmad

**Affiliations:** ^1^ Dasman Diabetes Institute Kuwait City Kuwait

**Keywords:** AhR, metabolic disease, obesity

## Abstract

**Background:**

The aryl hydrocarbon receptor (AhR) is linked to inflammation, but its plasma agonist activity and association with metabolic and inflammatory markers in obesity remain unclear. This cross‐sectional study aimed to determine the level of plasma AhR agonistic activity and its association with systemic inflammation and metabolic dysregulation in obesity.

**Methods:**

Plasma samples were collected from 80 non‐diabetic (39‐obese, 23‐overweight, and 18‐normal/healthy weight) individuals. AhR agonist activity was assessed using a cell‐based luciferase reporter assay. Plasma AhR was quantified by ELISA. Inflammatory markers were assessed using a multiplex Luminex platform.

**Results:**

Our findings indicate that plasma AhR agonist activity is elevated in obese (92.77 ± 4.002 fold activation) compared with normal/healthy weight (51.39 ± 2.335) and overweight participants (67.54 ± 5.24 fold activation). Moreover, the AhR protein was also elevated in obese (94.88 ± 7.62 pg/ml) compared to normal/healthy weight (65.88 ± 6.78 pg/ml) and overweight participants (67.54 ± 5.24 pg/ml), which was positively correlated with AhR activity (*r* = 0.441, *p* < 0.0001). AhR activity was positively correlated with inflammatory markers including IL‐1β, IL‐6, TNF‐α, TNF‐β, and MCP‐1, as well as metabolic markers such as BMI, total cholesterol, TG, insulin, FBG, and HbA1c. In contrast, it was negatively associated with HDL cholesterol. Notably, HOMA‐IR was positively correlated with AhR activity. In the regression model, TNF‐α, MCP‐1, BMI, and HDL cholesterol emerged as significant predictors of AhR activity.

**Conclusions:**

Our findings demonstrate that elevated plasma AhR agonist activity is associated with obesity, systemic inflammation, and metabolic dysregulation. These results highlight AhR activity as a biomarker of interest and support further studies to clarify its mechanistic role and potential clinical relevance in metabolic disorders.

## Graphical abstract

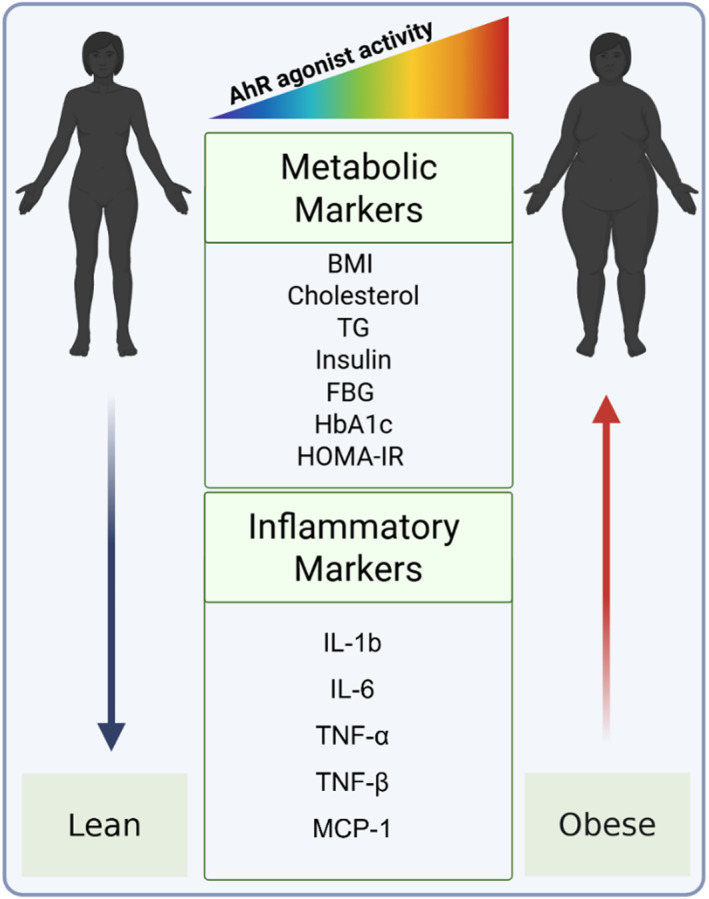

## 1 Introduction

Obesity is known as a global pandemic disorder and a major risk factor for hypertension, dyslipidemia, cardiovascular diseases (CVD), type 2 diabetes (T2D), metabolic dysfunction‐associated steatotic liver disease (MASLD), chronic kidney disease and certain cancers [1]. The World Obesity Atlas Federation concluded that more than 50% of the world's population would be overweight or obese within the next 12 years [2]. It is associated with a reduction in quality of life, shortened life span, and increased healthcare costs [3, 4]. Furthermore, although substantial progress has been made, the molecular mechanisms underlying obesity are not yet fully understood, and important knowledge gaps remain that limit a comprehensive understanding of its complex pathophysiology. The aryl hydrocarbon receptor (AhR) is a versatile transcription factor that plays a crucial role in sensing both exogenous and endogenous signals within the body. It was initially recognized for its role in the metabolism of xenobiotics, particularly compounds containing aromatic hydrocarbons [5]. AhR is now understood to have significant implications in various physiological processes, including inflammation and metabolic disorders such as diabetes [6]. AhR, which is part of the basic helix‐loop‐helix/per‐arnt‐sim (bHLH/PAS) superfamily, is expressed throughout many tissues and has conserved functions across multicellular organisms [7]. Upon binding to its ligands, AhR translocates from the cytoplasm to the nucleus, where it forms a complex with the AhR nuclear translocator (ARNT) [8]. This complex acts as a transcription factor that regulates genes associated with xenobiotic metabolism, particularly those in the cytochrome P450 family [9]. However, recent research has expanded our understanding of AhR's role beyond detoxification, highlighting its involvement in regulating inflammation and metabolic pathways [10, 11, 12].

Studies have demonstrated that AhR plays a pivotal role in the activation of inflammatory pathways, including the stimulation of mitogen‐activated protein kinases (MAPKs) and the nuclear factor‐kappa B (NF‐κB) signalling pathway, leading to the production of pro‐inflammatory cytokines [13, 14, 15]. Furthermore, elevated levels of AhR have been observed in individuals with obesity, suggesting a link between AhR signalling and metabolic dysregulation [16]. In experimental models, a high‐fat diet has been shown to increase vascular AhR expression, while inhibiting AhR has been linked to reduced vascular dysfunction associated with obesity [17]. Given this context, investigating the pathological relationship between AhR, inflammation, and obesity is essential for developing potential therapeutic strategies for metabolic disorders. Therefore, the current study aimed to investigate the role of plasma‐derived AhR agonistic activity in metabolic dysregulation among normal/healthy weight, overweight, and obese individuals and examine its associations with biomarkers of systemic inflammation and insulin resistance.

## 2 Material and Methods

### 2.1 Study Population and Anthropometric Measurements

A total of 80 non‐diabetic (39 obese, 23 overweight, and 18 normal/healthy weight) individuals were recruited in this study. The participants were classified as normal/healthy weight, overweight, and obese based on their body mass index (BMI). All participants gave written informed consent, and the study was approved by the ethics committee of Dasman Diabetes Institute, Kuwait (approval number RA 2010–2003). Height and weight were measured using calibrated portable electronic weighing scales and portable inflexible height measuring bars; the waist circumference was measured using a non‐stretchable tape at the midpoint between the lower margin of the last palpable rib and the top of the iliac crest, with participants standing and at the end of normal expiration, following standard anthropometric guidelines. BMI was calculated based on height and weight using standard formula: BMI = body weight (kg)/height (m^2^). The characteristics of the participants are summarised in Table 1.

**TABLE 1:** Anthropometric, clinical, and biochemical characteristics of the study participants.

Physical and biochemical characteristics	Normal/healthy weight, *n* = 18	OW + OB (*n* = 62)	Normal/healthy weight versus OW + OB
	Median (IQR)	Median (IQR)	*p*‐value
Age (years)	37.5 (28–51.25)	43.5 (33–51)	0.257
Weight (kg)	74.7 (60–82.7)	85.45 (71.58–99.23)	0.0048
Height (cm)	1.64 (1.59–1.70)	1.66 (1.58–1.76)	0.298
BMI (kg/m^2^)	23.39 (21.83–24.29)	33.06 (29.7–39.6)	< 0.0001
Waist circumference (cm)	78 (72–95)	98.5 (89.10–109.9)	0.0022
FBG (mmol/L)	4.995 (4.68–5.33)	5.0 (4.8–5.7)	0.3821
Triglycerides (mmol/L)	1.010 (0.42–1.94)	1.45 (0.92–2.06)	0.019
Total cholesterol (mmol/L)	4.8 (3.40–5.70)	5.41 (4.72–6.10)	0.023
HDL cholesterol (mmol/L)	1.09 (1.02–1.37)	1.18 (1.04–1.37)	0.389
LDL cholesterol (mmol/L)	2.70 (1.70–4.30)	3.35 (3.83–3.73)	0.004
Insulin (mU/L)	6.09 (4.96–16.86)	10.38 (5.34–46.08)	0.101
HbA1C (%)	5.55 (5.13–5.83)	5.70 (5.38–6.0)	0.331
HOMA‐IR	1.40 (1.07–6.30)	4.8 (1.30–12.09)	0.029

### 2.2 Measurement of Metabolic Markers

Peripheral blood was collected from overnight‐fasted individuals, and plasma was isolated by centrifugation at 1000 × g for 15 min at 4°C. Samples were subsequently analysed for fasting blood glucose (FBG), lipid profile, glycated haemoglobin (HbA1c), and fasting insulin. Glucose and lipid profiles (plasma triglycerides, HDL, and total cholesterol level) were measured using a Siemens Dimension RXL chemistry analyser (Diamond Diagnostics Holliston, MA). Glycated haemoglobin (HbA1c) was measured using a Variant device (BioRad, Hercules, CA, USA). HOMA‐IR as a measure of insulin resistance was calculated from basal (fasting) glucose and insulin concentrations using the following formula: HOMA‐IR = fasting insulin (μU/L) × fasting glucose (nmol/L)/22.5. All assays were performed following instructions from the manufacturers. The metabolic markers of the participants are summarised in Table 1.

### 2.3 Measurement of Aryl Hydrocarbon Receptor Activity

HepG2‐AhR Lucia reporter cells (InvivoGen, San Diego, CA) were used to evaluate the AhR activation. HepG2‐Lucia AhR reporter cells are genetically engineered from the human HepG2 liver carcinoma cell line. These cells express endogenous Aryl Hydrocarbon Receptor (AhR) and are stably transfected with a Lucia luciferase reporter gene driven by a minimal promoter containing the full CYP1A1 regulatory sequence with six dioxin‐responsive elements (DREs), enabling sensitive and specific detection of AhR‐mediated transcriptional activity. Cells were grown and maintained according to the manufacturer's instructions in Eagle's minimal essential medium, 10% FBS, 1X non‐essential amino acids medium, 100 μg/mL Normocin (InvivoGen), and 100 μg/mL Zeocin (Invivogen). The cell number and viability were assessed by exclusion of trypan blue dye using a haemacytometer. For the assay, HepG2‐Lucia AhR cells were seeded into 24‐well plates at a density of 1 × 10^5^ cells/well and incubated overnight to allow cell attachment. The following day, cells were co‐cultured with plasma samples at a final concentration of 50% derived from lean (normal weight), overweight, and obese individuals for 24 h.

After the incubation period, cell culture supernatants (conditioned media) were collected to quantify Lucia luciferase activity. The measurement was performed using QUANTI‐Luc 4 reagent (InvivoGen). Specifically, 20 μL of conditioned medium was combined with 50 μL of QUANTI‐Luc 4 reagent in each well of a 96‐well black plate. The plate was gently tapped several times to ensure proper mixing, and luminescence was measured immediately using a luminometer. The assay included a negative control (media only) to determine baseline luminescence and a positive control consisting of the known AhR agonist 6‐formylindolo[3,2‐b]carbazole (FICZ) to confirm assay responsiveness. A vehicle control (0.1% DMSO) was included in all experiments. To assess specificity, cells were co‐treated with the AhR antagonist CH‐223191 (1 μM), confirming that reporter activation was AhR‐dependent. Reporter activity was normalised to cell viability using the MTT assay and cell counts to ensure that differences reflected changes in AhR activity rather than variations in cell number or viability. Experiments were performed in (triplicate wells) and repeated independently (3 times).

### 2.4 Measurement of Aryl Hydrocarbon Receptor

Human Aryl Hydrocarbon Receptor (AhR) protein levels in participant plasma samples were quantified using a commercially available ELISA kit (Cat. # CSB‐E09355h, CUSABIO, USA) following the manufacturer's instructions. Samples and standards were assayed in triplicates, and a standard curve was generated using serial dilutions of the provided standard to determine concentrations. Prior to the assay, plasma samples were pre‐cleared by centrifugation at 10,000 × g for 10 min to remove debris and lipids. Samples were then diluted appropriately to ensure measurements fell within the linear dynamic range of the assay.

### 2.5 Human XL Cytokine Luminex Performance Assay

A Luminex‐based multiplex assay was performed to simultaneously quantify 46 cytokines and chemokines in human plasma samples. The assay utilised the Human XL Cytokine Luminex Performance Assay 46‐plex Fixed Panel (Catalogue No. LKTM014B, Bio‐Techne, R&D Systems, Minneapolis, MN, USA) and was conducted according to the manufacturer's protocol.

The panel included the following analytes: CD40 Ligand, EGF, Eotaxin, FGF basic, G‐CSF, GM‐CSF, Granzyme B, GROα, GROβ, IFN‐α2, IFN‐β, IFN‐γ, IL‐1α, IL‐1β, IL‐1ra, IL‐2, IL‐3, IL‐4, IL‐5, IL‐6, IL‐7, IL‐8, IL‐9, IL‐10, IL‐12p70, IL‐13, IL‐15, IP‐10, MCP‐1, MIP‐1α, MIP‐1β, MIP‐3α, MIP‐3β, PDGF‐AA, PDGF‐AA/BB, RANTES, TGF‐α, TNF‐α, TNF‐β, TRAIL, and VEGF.

Plasma samples were assayed in a 96‐well plate format, and readings were obtained using a Bio‐Rad Bio‐Plex analyser. All samples were analysed in duplicate, and standard curves for each analyte were included to ensure accurate quantification.

### 2.6 Statistical Analysis

Statistical analyses were performed using GraphPad Prism (version 13, GraphPad Software, La Jolla, CA, USA). Data distribution was assessed for normality using the Shapiro–Wilk and Kolmogorov–Smirnov tests. As the majority of variables did not follow a normal distribution, data are presented as medians with interquartile ranges (25th–75th percentiles).

Comparisons between the two groups were performed using the Mann–Whitney *U* test. For comparisons among three or more groups, the Kruskal–Wallis test followed by Dunn's corrected post hoc multiple comparison test was applied. Correlations between Ahr activity/protein levels and metabolic and inflammatory markers were assessed using Spearman's rank correlation coefficient. Data are presented as mean ± standard error of mean (SEM) *p* value < 0.05 was considered statistically significant.

## 3 Results

### 3.1 Demographic Data, Anthropometric Measurements, Insulin Resistance, and Lipid Profile

Clinical and biochemical characteristics of normal/healthy weight, overweight and obese are shown in Table 1. All groups were age matched. Overweight/Obese individuals had a significantly higher weight (*p* = 0.0048), BMI (*p* < 0.0001), cholesterol (*p* = 0.023) and waist circumference (*p* = 0.0022) compared to the normal/healthy weight groups.

### 3.2 Plasma AhR Protein Levels are Significantly Increased in Obesity

**FIGURE 1:**
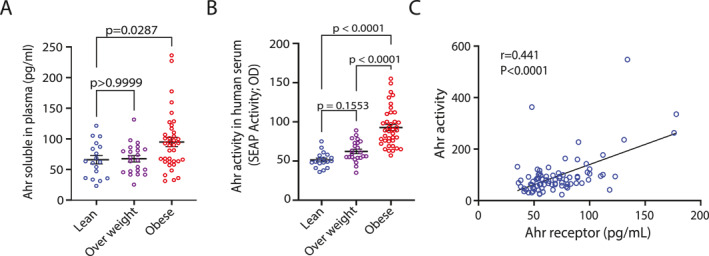
AhR protein levels and AhR agonist activity in normal/healthy weight, overweight and obese plasma samples. (A): AhR plasma protein levels of healthy normal/healthy weight, overweight and obese were assessed in triplicate (*n* = 3) using Human Aryl Hydrocarbon Receptor ELISA Kit. (B): AhR agonist activity in plasma samples of normal/healthy weight (*n* = 18), overweight (*n* = 23) and obese (*n* = 39), was assessed in technical duplicates using an AhR ligand sensitive luciferase assay. (C): Solid line shows linear regression with correlation of AhR activity and AhR soluble protein in plasma. Values represent the actual measurements of each sample. Lines represent mean and error bar SEM. For comparisons among three or more groups, the Kruskal–Wallis test followed by Dunn's corrected post hoc multiple comparison test was applied. *p* < 0.05 was considered significant.

Since inhibiting the aryl hydrocarbon receptor reverses obesity and hepatic steatosis in mice by modulating genes related to lipid metabolism and inflammation [18], we investigated whether AhR levels are altered in human obesity. To address this, we measured plasma AhR protein levels in normal/healthy weight, overweight, and obese nondiabetic individuals. Plasma AhR levels were significantly elevated in the obese group compared to both the normal/healthy weight and overweight groups (Figure 1A).

### 3.3 Plasma AhR Agonist Activity is Elevated in Obesity

Our data show that plasma AhR is elevated in obesity. Next, we asked whether this increase affects plasma AhR agonist activity. To investigate this, plasma samples were tested in vitro for AhR activation using the HepG2–AhR–Luc cell line, where luciferase expression depends on AhR activity. Luminescence signals (Figure 1B) revealed that plasma from obese individuals induced significantly higher AhR agonistic activity compared to samples from normal/healthy weight, and overweight individuals (*p* < 0.0001). Interestingly, AhR activity showed a strong and statistically significant positive correlation with soluble AhR protein levels in plasma samples (*r* = 0.441, *p* < 0.0001) (Figure 1C).

### 3.4 Plasma AhR Agonist Activity is Correlated With Inflammatory Markers

**FIGURE 2:**
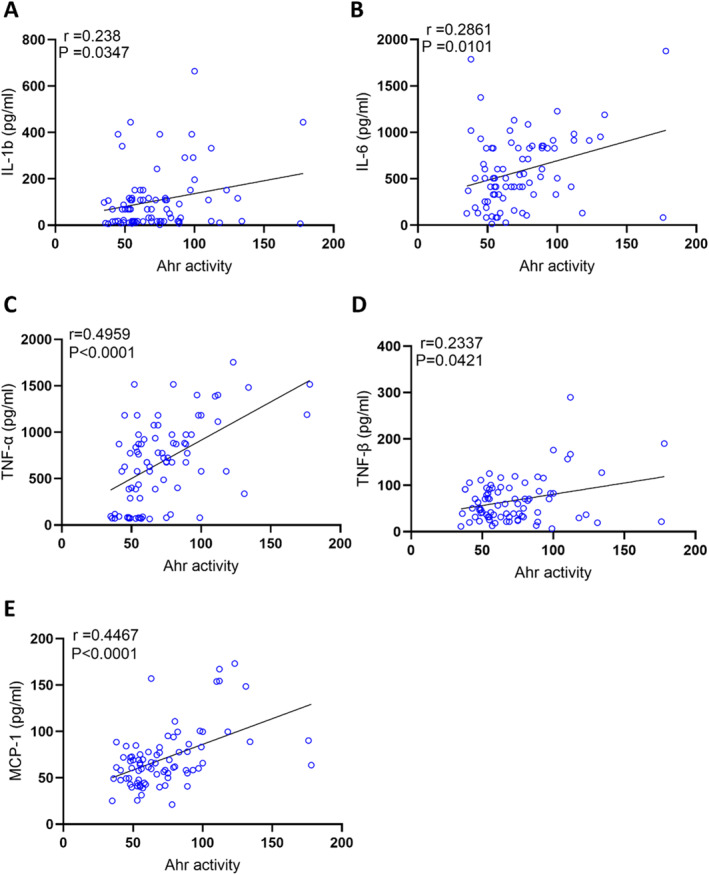
AhR agonist activity in healthy plasma samples was assessed in technical duplicates using an AhR ligand sensitive luciferase assay. AhR activity levels of healthy participants were positively correlated with IL‐1β (A), IL‐6 (B), TNF‐α (C) TNF‐β (D), MCP1 (E). Values represent the actual measurements of each sample. Significance levels were determined using comparisons between the two groups using the Mann–Whitney *U* test. For comparisons among three or more groups, the Kruskal–Wallis test followed by Dunn's corrected post hoc multiple comparisons test was applied. Correlations between Ahr activity/protein levels and metabolic and inflammatory markers were assessed using Spearman's rank correlation coefficient. *p*‐value < 0.05 was considered statistically significant.

AhR has been documented to play a role in inflammation [6, 19, 20]. Based on the results presented in Table 2, we hypothesised that increased AhR agonist activity in obesity may influence inflammatory markers. To test this, we measured plasma levels of inflammatory markers in the same samples used to assess AhR agonist activity. Our data show that plasma AhR agonist activity is positively correlated with inflammatory cytokines (IL‐1b, IL‐6, TNF‐α, TNF‐β) (Figure 2A–D). Other cytokines including IL‐1aIL‐2, IL‐3, IL‐4, IL‐5, IL‐7, IL‐8, IL‐10, IL‐13, IL‐15 and IL‐33 did not show significant associations. However, there is no association that was found between AhR agonist activity and interferons (IFN‐α, IFN‐β, IFN‐γ). Plasma AhR agonist activity showed a strong correlation with MCP‐1 (*r* = 0.4467, *p* < 0.0001) (Figure 2E), highlighting a potential link with monocyte recruitment and low‐grade systemic inflammation. Chemokines (MIP‐1α, MIP‐1β, MIP‐3a, MIP‐3b, IP‐10, RANTES) did not show significant association with AhR agonist activity.

**TABLE 2:** Correlation of plasma AhR agonist activity with inflammatory markers.

Cytokines	Spearman *r*‐value	95% confidence interval	*p*‐value	*p*‐value summary
IL‐1a	0.0911	−0.1392 to 0.3121	0.4246	ns
IL‐b	0.238	0.01116 to 0.4415	0.0347	*
IL‐1ra	0.2374	0.002522 to 0.4474	0.0417	*
IL‐2	0.08405	−0.1447 to 0.3043	0.4585	ns
IL‐3	−0.05003	−0.2729 to 0.1780	0.6594	ns
IL‐4	0.05146	−0.1781 to 0.2757	0.6525	ns
IL‐5	−0.02042	−0.2453 to 0.2065	0.8573	ns
IL‐6	0.2861	0.06425 to 0.4810	0.0101	*
IL‐7	0.004114	−0.2221 to 0.2299	0.9711	ns
IL‐8	0.04739	−0.1820 to 0.2719	0.6784	ns
IL‐10	0.006481	−0.2198 to 0.2321	0.9545	ns
IL‐13	0.09715	−0.1317 to 0.3162	0.3913	ns
IL‐15	0.04437	−0.1835 to 0.2677	0.696	ns
IL‐17a	−0.07365	−0.2947 to 0.1549	0.5162	ns
IL‐33	−0.09333	−0.3127 to 0.1355	0.4103	ns
TNF‐α	0.4959	0.3039 to 0.6491	< 0.0001	****
TNF‐β	0.2337	0.001968 to 0.4417	0.0421	*
Chemokines				
MCP‐1	0.4467	0.2455 to 0.6110	< 0.0001	****
IP‐10	−0.06187	−0.2839 to 0.1664	0.5856	ns
MIP‐1a	−0.2969	−0.4900 to −0.07605	0.0075	**
MIP‐1b	−0.02233	−0.2471 to 0.2047	0.8442	ns
MIP‐3a	0.09525	−0.1336 to 0.3145	0.4007	ns
MIP‐3b	0.1151	−0.1138 to 0.3325	0.3093	ns
Rantes	−0.08766	−0.3076 to 0.1411	0.4394	ns
Interferons				
IFNα	0.07412	−0.1545 to 0.2952	0.5135	ns
IFNβ	−0.1457	−0.3599 to 0.08300	0.1971	ns
IFNγ	−0.03353	−0.2576 to 0.1939	0.7678	ns

### 3.5 Association of Plasma AhR Agonist Activity With Clinical Metabolic Markers

**FIGURE 3:**
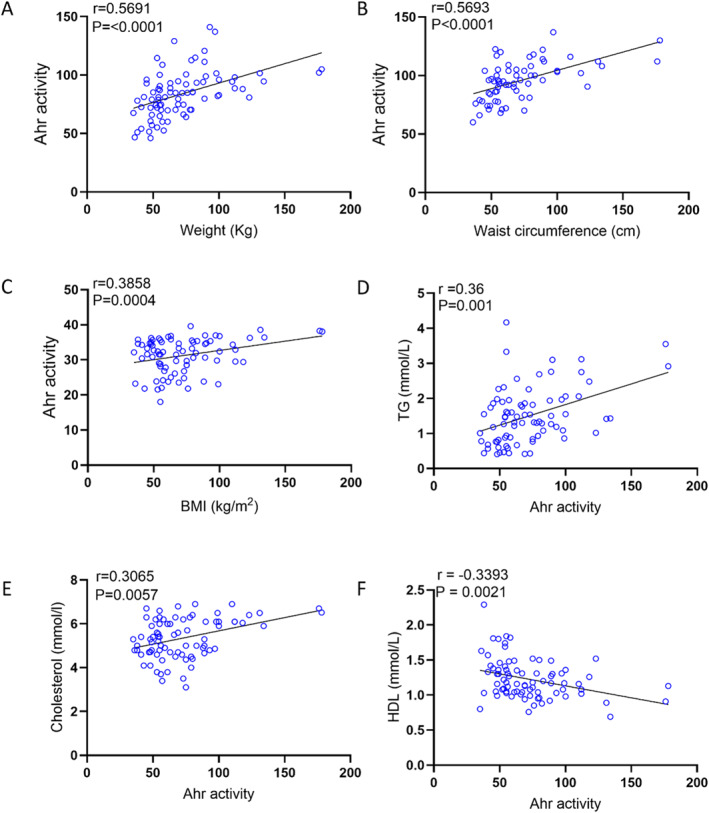
AhR agonist activity in healthy plasma samples with clinical markers was assessed in technical duplicates using an AhR ligand sensitive luciferase assay. AhR activity levels of healthy participants were positively correlated with weight (A), waist circumference (B), BMI (C), TG (D), Cholesterol (E), and HDL (F). Values represent the actual measurements of each sample. Correlations between Ahr activity levels and metabolic markers were assessed using Spearman's rank correlation coefficient, *p* < 0.05 was considered significant.

**FIGURE 4:**
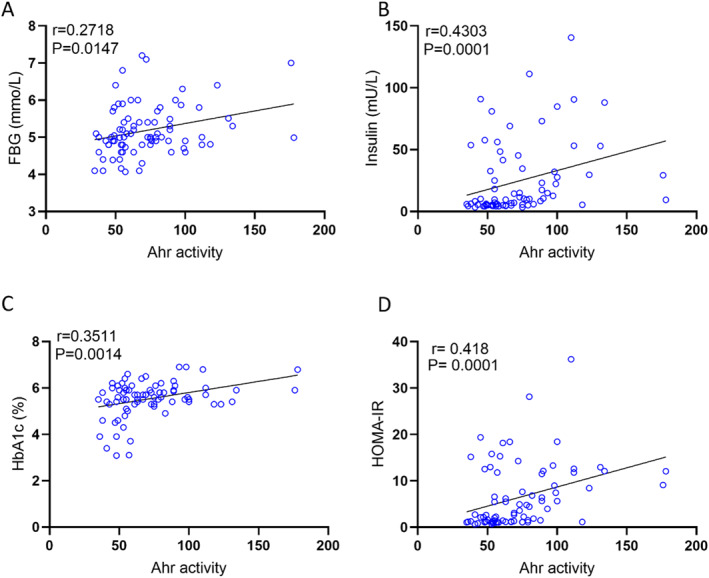
AhR agonist activity in healthy plasma samples with insulin sensitivity markers was assessed in technical duplicates using an AhR ligand sensitive luciferase assay. AhR activity levels of healthy participants were positively correlated with FBG (A), insulin (B), HbA1C (C), and HOMA‐IR (D). Values represent the actual measurements of each sample. Correlations between Ahr activity levels and metabolic markers were assessed using Spearman's rank correlation coefficient, *p* < 0.05 was considered significant.

Next, we asked whether the changes in plasma AhR agonist activity were associated with clinico‐metabolic signatures. To this end, we measured serum levels of triglycerides, total cholesterol, high density lipoprotein (HDL) cholesterol, low density lipoprotein (LDL) cholesterol, fasting blood glucose (FBG), HbA1c, insulin, and CRP as shown in Table 3. Plasma AhR agonist activity was found to be positively associated with weight (*r* = 0.5691, *p* < 0.0001), waist circumference (*r* = 0.5693, *p* < 0.0001), and BMI (*r* = 0. 3858, *p* = 0. 0004) (Figure 3A–C). Additionally, AhR activity was positively correlated with triglycerides (TG) (*r* = 0.36, *p* = 0.001) and total cholesterol (Chol) (*r* = 0.3065, *p* = 0.0057) (Figure 3D–E). However, HDL cholesterol was negatively correlated with AhR agonist activity (Figure 3F). Of note, AhR agonist activity expression showed trend positive correlation with levels of systemic inflammatory marker CRP (*r* = 0.2432, *p* = 0.0709). Furthermore, AhR agonist activity was positive for FBG (*r* = 0.2718, *p* = 0.0147), insulin (*r* = 0.4304, *p* = 0.0001) and HbA1c (*r* = 0.3511, *p* = 0.0014) (Figure 4A–C). Finally, insulin resistance high homoeostatic model assessment of insulin resistance (HOMA‐IR) correlated with elevated plasma AhR agonist activity (Figure 4D).

**TABLE 3:** AhR activity of participants plasma correlation with clinical and biochemical characteristics.

	Spearman *r*‐value	95% confidence interval	*p*‐value	*p*‐value summary
Ahr protein	0.2596	0.03565 to 0.4586	0.0201	*
Age	0.2396	0.01434 to 0.4416	0.0323	*
Height (m)	0.1335	−0.09537 to 0.3490	0.2378	ns
Weight (Kg)	0.5691	0.3937 to 0.7045	< 0.0001	****
Waist	0.5693	0.3715 to 0.7176	< 0.0001	****
BMI	0.3858	0.1751 to 0.5628	0.0004	***
Chol (mmoL/L)	0.3065	0.08649 to 0.4980	0.0057	**
HDL (mmoL/l)	−0.3393	−0.5250 to −0.1227	0.0021	**
LDL (mmoL/l)	0.1577	−0.07086 to 0.3704	0.1625	ns
TGL (mmoL/l)	0.36	0.1459 to 0.5419	0.001	**
CRP	0.2432	−0.02904 to 0.4818	0.0709	ns
Insulin (mU/L)	0.4304	0.2206 to 0.6022	0.0001	***
HOMA‐IR	0.418	0.2105 to 0.5894	0.0001	***
FBG (mmoL/L)	0.2718	0.04885 to 0.4690	0.0147	*
HbA1c (%)	0.3511	0.06926 to 0.4848	0.0014	**

### 3.6 Plasma AhR Agonist Activity is Independently Associated With Inflammatory and Metabolic Dysregulation

Multiple linear regression analysis was conducted to determine the independent predictors of AhR agonist activity. The overall model was statistically significant (*R* ^2^ = 0.517, adjusted *R* ^2^ = 0.484, *p* < 0.001), indicating that approximately 51.7% of the variance in AhR agonist activity could be explained by the included inflammatory and metabolic markers (Table 4). Among the predictors, TNF‐α (*β* = 0.294, *p* = 0.002), MCP1 (*β* = 0.270, *p* = 0.005), and IL‐1ra (*β* = 0.270, *p* = 0.002) were significantly and positively associated with AhR agonist activity, highlighting their potential role in the pro‐inflammatory modulation of AhR signalling. In addition, BMI (*β* = 0.193, *p* = 0.024) was positively associated with AhR agonist activity, whereas HDL (*β* = −0.191, *p* = 0.029) showed a significant inverse association (Table 4). These findings suggest that elevated AhR activity is independently associated with systemic inflammation and metabolic dysregulation.

**TABLE 4:** Regression model shows independent predictors of plasma AhR agonist activity.

Multiple linear regression—model summary			
R	*R* ^2^	Adjusted *R* ^2^	Anova (sig)
0.719	0.517	0.484	< 0.001

Dependent variable	Predictor variables	Standardized coefficient (β–value)	*p*‐value
AhR activity	MCP1	0.270	0.005
	TNF‐α	0.294	0.002
	HDL	−0.191	0.029
	BMI	0.193	0.024
	IL‐1ra	0.270	0.002

## 4 Discussion

In this cross‐sectional study, we report that plasma aryl hydrocarbon receptor (AhR) protein levels and agonist activity are significantly elevated in individuals with obesity. Our data demonstrate that AhR activity is positively associated with multiple inflammatory and metabolic markers, supporting the hypothesis that AhR plays a key role in linking low‐grade chronic inflammation to metabolic dysregulation in human obesity. These findings align with and extend previous preclinical studies by providing human‐based evidence of AhR involvement in obesity‐related immunometabolic alterations.

Our observation that plasma AhR protein levels are elevated in obesity is consistent with earlier findings in animal models where AhR contributes to obesity and hepatic steatosis through modulation of genes involved in lipid metabolism and inflammation [21]. Importantly, we extend these findings by showing that not only AhR protein but also AhR agonist activity is significantly increased in the plasma of obese individuals, suggesting an upregulation of both receptor abundance and its activation state. The use of a luciferase reporter assay (HepG2–AhR–Luc cell line) provided functional evidence of AhR activation, which was strongly correlated with soluble AhR protein levels. A key finding of this study is a strong positive association between AhR agonist activity and systemic concentrations of inflammatory cytokines and chemokines, notably IL‐1β, IL‐6, TNF‐α, TNF‐β, and MCP‐1. Notably, multivariate regression confirmed that TNF‐α and MCP‐1 were independently and significantly associated with AhR activity, suggesting a central role in the inflammatory response. These cytokines are well‐established mediators of chronic low‐grade inflammation in obesity [22], and our results suggest that they may contribute to or result from AhR activation in the systemic circulation. Notably, these findings are supported by prior work indicating that AhR activation in immune cells enhances pro‐inflammatory cytokine expression, including TNF‐α and IL‐1 family members [23, 24]. Increased AhR transcript levels were positively correlated with the frequencies of pro‐inflammatory Th17, Th22, and Th1 cells, as well as circulating levels of IL‐22 and IL‐17, highlighting a link between AhR activation and systemic inflammation in metabolic disease [25]. Interestingly, while AhR agonist activity was associated with several pro‐inflammatory cytokines and metabolic disturbances, no significant association was observed with interferons or a range of chemokines, suggesting a selective pattern of immune modulation. This specificity may reflect the distinct transcriptional pathways engaged by AhR under different ligand contexts, as has been observed in immune cell differentiation studies [26].

In addition to inflammation, metabolic markers were significantly associated with plasma AhR activity, including positive correlations with BMI, waist circumference, triglycerides, total cholesterol, fasting glucose, insulin, HbA1c, and HOMA‐IR as well as a negative correlation with HDL cholesterol. These associations remained in multiple linear regression, where BMI emerged as a significant independent predictor, and HDL retained an inverse relationship. This pattern suggests that elevated AhR activation is closely associated with a broader metabolic stress signature. Prior studies in murine models have shown that AhR antagonism improves glucose metabolism and lipid profiles [27], supporting a link between AhR activity and metabolic regulation. Similarly, other research in individuals with T2DM has shown a positive association between serum AhR ligand activity and insulin resistance, a hallmark of T2DM, attributed to disrupted glucose homoeostasis and impaired insulin signalling triggered by AhR activation [28]. However, these observations are inherently associative and do not establish a causal role for AhR activation in metabolic dysfunction. In addition, prior human studies have largely focused on diabetic populations and have not simultaneously evaluated inflammatory and metabolic parameters. In contrast, our study provides an integrated assessment of inflammatory cytokines and metabolic biomarkers in non‐diabetic individuals across a range of body mass indices (normal/healthy weight, overweight, and obese). Notably, our findings show that elevated AhR agonist activity is detectable in obesity prior to the onset of overt diabetes and is consistently associated with markers of systemic inflammation and metabolic perturbation. Although these results do not imply causality, they suggest that AhR agonist activity may serve as a useful indicator of early obesity‐associated immunometabolic alterations.

Given that insulin resistance was present across participants and some individuals had prediabetes, it is important to consider how these metabolic states may have influenced our findings. Importantly, a proportion of participants in our cohort exhibited prediabetes, and all individuals were characterised by insulin resistance, which may have influenced the observed associations. Insulin resistance is known to promote chronic low‐grade inflammation, dysregulated lipid metabolism, and altered production of endogenous metabolites, including tryptophan‐derived compounds that can serve as AhR ligands [29, 30, 31]. Thus, the elevated AhR activity observed in this study may reflect, at least in part, the underlying insulin‐resistant state rather than obesity alone. Prediabetes represents an intermediate stage of metabolic dysfunction characterised by impaired glucose regulation and heightened inflammatory signalling, which could further amplify AhR activation and its associations with cytokines and metabolic markers [32, 33, 34]. While our study focused on non‐diabetic individuals, the presence of insulin resistance highlights the possibility that AhR signalling may be responsive to early metabolic disturbances along the continuum from obesity to diabetes. Future studies stratifying participants by insulin sensitivity and glycaemic status will be important to disentangle the independent contributions of obesity, insulin resistance, and prediabetes to systemic AhR activation.

This study has some limitations that should be considered when interpreting the findings. Most notably, the cross‐sectional design precludes the establishment of causal relationships between AhR agonist activity and obesity‐associated inflammatory and metabolic alterations. While the observed associations support a link between enhanced AhR activation and immunometabolic dysfunction in obesity, they do not allow conclusions regarding the temporal sequence or directionality of these effects. Longitudinal studies are therefore warranted to determine whether elevated AhR agonist activity precedes the development of metabolic impairment or arises as a consequence of obesity‐related inflammation. Furthermore, mechanistic and interventional studies will be essential to clarify the causal role of AhR signalling in metabolic dysregulation and to evaluate its potential as an early biomarker or therapeutic target in obesity.

## 5 Conclusion

Our study shows that plasma AhR agonist activity is independently associated with key inflammatory cytokines and metabolic markers in individuals with obesity. These associations support the relevance of AhR activation as an integrative indicator of obesity‐associated immunometabolic alterations, even in the absence of overt diabetes.

## Author Contributions

F.B, S.K, A.A participated in performing experiments and collecting and analysing data. F.B wrote the manuscript. A.M participated in analysing and writing the manuscript. A.B, M.A, F.A reviewed and critically commented on the manuscript and participated in interpretation of the data, and R.A. conceived the idea, guided the research study, provided material support, procured funds, wrote, edited, and approved the manuscript for submission.

## Conflicts of Interest

The authors declare no conflicts of interest.

## Data Availability

The data that support the findings of this study are available from the corresponding author upon reasonable request.
